# Supplementary material for: Scores for sepsis detection and risk stratification – construction of a novel score using a statistical approach and validation of RETTS
Source: PLoS One. 2020 Feb 20;15(2):e0229210. doi: 10.1371/journal.pone.0229210 (PMC7032705; doi:10.1371/journal.pone.0229210)
Supplement: S1 Table — (DOCX) [file pone.0229210.s002.docx]

**Table I. RETTS**

|  | Red | Orange | Yellow | Green |
| --- | --- | --- | --- | --- |
| A | Blocked airway or stridor |  |  |  |
| B | Respiratory rate >30 or <8  SaO_2_<90 with oxygen (O_2_) | Respiratory rate >25  SaO_2_<90 without O_2_ | SaO_2_≤95 | SaO_2_>95 without O_2_ |
| C | Heart rate >130 if sinus rythm, else >150  SBP <90 | Heart rate >120 or <40 | Heart rate >110 or <50 | Heart rate 50-110 |
| D | Unconscious or cramps | Somnolence | Acute disorientation | Alert |
| E |  | Temperature >41˚or <35˚ | Temperature >38.5˚ |  |
