# Supplementary material for: Scores for sepsis detection and risk stratification – construction of a novel score using a statistical approach and validation of RETTS
Source: PLoS One. 2020 Feb 20;15(2):e0229210. doi: 10.1371/journal.pone.0229210 (PMC7032705; doi:10.1371/journal.pone.0229210)
Supplement: S2 Table — (DOCX) [file pone.0229210.s003.docx]

**Table II. Organ dysfunction definition.**

| System | Values |
| --- | --- |
| Cardiovascular | SBP <90 mmHg  ∆SBP > -40  MAP <70  Vasopressor |
| Respiratory, cohort A | SaO_2_ <90% |
| Respiratory, cohort B | SaO_2_ <90%  For COPD SaO_2_ <87%  For SaO_2_ 90-94% and O_2_ PaO_2_/FiO_2_ ratio < 300  with COPD and SaO_2_ 87-95% PaO_2_/FiO_2_ ratio < 250 |
| Renal | creatinine increase > 44 µmol/L  urinary output < 0.5mL/kg/h for > 2 hours  initiation of dialysis |
| Hepatic | bilirubin > 35μmol/L |
| Hematologic | platelet count < 100 x 10^9^/L  INR > 1.5 unless use of anticoagulants |
| Metabolic | lactate > 3.2 mmol/L |
