# Supplementary material for: Scores for sepsis detection and risk stratification – construction of a novel score using a statistical approach and validation of RETTS
Source: PLoS One. 2020 Feb 20;15(2):e0229210. doi: 10.1371/journal.pone.0229210 (PMC7032705; doi:10.1371/journal.pone.0229210)
Supplement: S3 Table — (DOCX) [file pone.0229210.s004.docx]

**Table III. NEWS2 score**

|  | 3 | 2 | 1 | 0 | 1 | 2 | 3 |
| --- | --- | --- | --- | --- | --- | --- | --- |
| Respiration rate | <9 |  | 9-11 | 12-20 |  | 21-24 | >24 |
| SpO_2_ Scale 1 (%) | <92 | 92-93 | 94-95 | >95 |  |  |  |
| SpO_2_ Scale 1 (%) | <84 | 84-85 | 86-87 | 88-92  >92 on air | 93-94 on oxygen | 95-96 on oxygen | >96 on oxygen |
| Air or Oxygen |  | Oxygen |  | Air |  |  |  |
| SBP (mmHg) | <91 | 91-100 | 101-110 | 111-219 |  |  | >219 |
| Pulse | <41 |  | 41-50 | 51-90 | 91-110 | 111-130 | >130 |
| Consciousness |  |  |  | Alert |  |  | VPCU |
| Temperature (˚C) | <35.1 |  | 35.1-36.0 | 36.1-38.0 | 38.1-39.0 | >39.0 |  |
