# Supplementary material for: Scores for sepsis detection and risk stratification – construction of a novel score using a statistical approach and validation of RETTS
Source: PLoS One. 2020 Feb 20;15(2):e0229210. doi: 10.1371/journal.pone.0229210 (PMC7032705; doi:10.1371/journal.pone.0229210)
Supplement: S4 Table — (DOCX) [file pone.0229210.s005.docx]

**Table IV. Missing data.**

|  | **Cohort A** | | | **Cohort B** | | |  | **Validation cohort** |  |
| --- | --- | --- | --- | --- | --- | --- | --- | --- | --- |
|  | n | % | n | | % |  |  |  |  |
| **Systolic Blood Pressure** | 4 | <1 | 4 | | <1 |  |  |  |  |
| **Diastolic Blood Pressure** | 8 | 1 | 15 | | 3 |  |  |  |  |
| **Heart Frequency** | 1 | <1 | 3 | | <1 |  |  |  |  |
| **SaO2** | 10 | 2 | 14 | | 3 |  |  |  |  |
| **Respiratory Frequency** | 7 | 1 | 14 | | 3 |  |  |  |  |
| **Temperature** | 8 | 1 | 17 | | 4 |  |  |  |  |
| **Mental Status** | 6 | 1 | 4 | | <1 |  |  |  |  |
| **Lactate** | 32 | 6 | 185* | | 38* |  |  |  |  |
| **HBP** | 18 | 3 | 0 | | 0 |  |  |  |  |
| **Age** | 3 | <1 | 0 | | 0 |  |  |  |  |
| **Oxygen treatment** | 6 | 1 | 0 | | 0 |  |  |  |  |

* Lactate was not included in any analyses in Cohort B and hence not imputated
