# Supplementary material for: Scores for sepsis detection and risk stratification – construction of a novel score using a statistical approach and validation of RETTS
Source: PLoS One. 2020 Feb 20;15(2):e0229210. doi: 10.1371/journal.pone.0229210 (PMC7032705; doi:10.1371/journal.pone.0229210)
Supplement: S5 Table — (DOCX) [file pone.0229210.s006.docx]

**Table V. Demographics and outcome of patients with missing data.**

|  | Cohort A | | | Cohort B | | |
| --- | --- | --- | --- | --- | --- | --- |
|  | Without sepsis  n = 28 | | With sepsis  n = 21 | Without sepsis  n=39 | With sepsis  n= 15 | |
| Age, median | 57 | 70 | | 70 | | 73 |
| Female, n (%) | 12 (43) | 11 (52) | | 23 (59) | | 6 (40) |
|  |  |  | |  | |  |
| Comorbidities n (%) |  |  | |  | |  |
| Diabetes | 1 (4) | 4 (19) | | 8 (21) | | 5 (33) |
| Cardiovascular disease | 6 (21) | 7 (33) | | 17 (44) | | 7 (47) |
| Renal Disease | 1 (4) | 2 (10) | | 6 (15) | | 4 (27) |
| Liver Disease | 1 (4) | 0 (0) | | 0 (0) | | 1 (7) |
| Malignancy | 2 (7) | 4 (19) | | 4 (10) | | 2 (13) |
| Immunodeficiency | 2 (7) | 1 (5) | | 0 (0) | | 0 (0) |
| Respiratory Disease | 2 (7) | 2 (9) | | 6 (15) | | 2 (13) |
| No comorbidities | 18 (64) | 8 (38) | | 16 (41) | | 4 (27) |
| Organ dysfunction, n (%) |  | 20 (95) | | 22 (56) | | 15 (100) |
| Neurologic |  | 2 (9) | | 20 (51) | | 6 (40) |
| Cardiovascular |  | 15 (71) | | 14 (36) | | 14 (93) |
| Respiratory |  | 3 (14) | | 15 (39) | | 7 (47) |
| Renal |  | 5 (24) | | 2 (5) | | 5 (33) |
| Hematological |  | 4 (19) | | 2 (5) | | 1 (7) |
| Hepatic |  | 3 (14) | | 0 (0) | | 0 (0) |
|  |  |  | |  | |  |
| Intensive Care n (%) |  | 2 (9) | | 10 (26) | | 3 (20) |
| 3-days mortality n (%) |  | 1 (5) | | 3 (8) | | 0 (0) |

**Multiple imputation**

Variables imputed for cohort A were systolic blood pressure, diastolic blood pressure, heart frequency, respiratory frequency, temperature, mental status, SaO_2_, oxygen treatment, lactate, age and HBP. Other parameters in the imputation although not imputed were comorbidities and outcome. Predictive Mean Matching were used for multiple imputation and logistic regression for binary variables. The models were validated by plots of imputations and iterations.

Variables imputed for cohort B were systolic blood pressure, diastolic blood pressure, heart frequency, respiratory frequency, temperature, mental status, SaO_2_, oxygen treatment, lactate. Other parameters in the imputation although not imputed were comorbidities, outcome age and HBP. Predictive Mean Matching were used for multiple imputation and logistic regression for binary variables. The models were validated by plots of imputations and iterations.
