# Supplementary material for: Scores for sepsis detection and risk stratification – construction of a novel score using a statistical approach and validation of RETTS
Source: PLoS One. 2020 Feb 20;15(2):e0229210. doi: 10.1371/journal.pone.0229210 (PMC7032705; doi:10.1371/journal.pone.0229210)
Supplement: S6 Table — (DOCX) [file pone.0229210.s007.docx]

**Table VI. AUC for different risk stratification scores for sepsis, missing values were substituted by multiple imputation**

|  | **Cohort A** | | | **Cohort B** | | |
| --- | --- | --- | --- | --- | --- | --- |
|  | **AUC** | **95% CI** | ***p*** | **AUC** | **95% CI** | ***p*** |
| **NEWS2** | 0.79 | 0.75-0.83 | reference | 0.68 | 0.63-0.74 | reference |
| **RETTS** | 0.74 | 0.69-0.77 | 0.04 | 0.53 | 0.48-0.59 | <0.01 |
| **SEWS** | * | * | * | 0.67 | 0.62-0.73 | 0.80 |
| **SHEWS** | * | * | * | 0.74 | 0.69-0.79 | 0.06 |
|  |  |  |  |  |  |  |

* Not able to validate, derived in this cohort
