# Supplementary material for: Scores for sepsis detection and risk stratification – construction of a novel score using a statistical approach and validation of RETTS
Source: PLoS One. 2020 Feb 20;15(2):e0229210. doi: 10.1371/journal.pone.0229210 (PMC7032705; doi:10.1371/journal.pone.0229210)
Supplement: S7 Table — (DOCX) [file pone.0229210.s008.docx]

**Table VII. AUC for different risk stratification scores detection of combined outcome for sepsis compared to sepsis-3 definition, cohort A**

|  | **Combined outcome** | | | **Sepsis-3** | | |  |  |
| --- | --- | --- | --- | --- | --- | --- | --- | --- |
|  | **AUC** | **95% CI** | **AUC** | | | **95% CI** | | |
| **NEWS2** | 0.84 | 0.80-0.88 | 0.87 | | | 0.84-0.90 | | |
| **RETTS** | 0.76 | 0.71-0.81 | 0.78 | | | 0.74-0.82 | | |
| **SEWS** | 0.85 | 0.82-0.89 | 0.81 | | | 0.77-0.85 | | |
| **SHEWS** | 0.86 | 0.82-0.89 | 0.79 | | | 0.75-0.83 | | |
|  |  |  |  | |  | | |  |
