# Supplementary material for: Scores for sepsis detection and risk stratification – construction of a novel score using a statistical approach and validation of RETTS
Source: PLoS One. 2020 Feb 20;15(2):e0229210. doi: 10.1371/journal.pone.0229210 (PMC7032705; doi:10.1371/journal.pone.0229210)
Supplement: S8 Table — (DOCX) [file pone.0229210.s009.docx]

**Table VIII. AUC for different risk stratification scores prediction of combined outcome for sepsis compared to sepsis-3 definition, cohort B**

|  | **Combined outcome** | | **Sepsis-3** | |
| --- | --- | --- | --- | --- |
|  | **AUC** | **95% CI** | **AUC** | **95% CI** |
| **NEWS2** | 0.69 | 0.63-0.74 | 0.79 | 0.74-0.84 |
| **RETTS** | 0.55 | 0.49-0.60 | 0.60 | 0.54-0.66 |
| **SEWS** | 0.67 | 0.61-0.73 | 0.74 | 0.69-0.80 |
| **SHEWS** | 0.73 | 0.68-0.79 | 0.77 | 0.74-0.84 |
|  |  |  |  |  |
