# Supplementary material for: Scores for sepsis detection and risk stratification – construction of a novel score using a statistical approach and validation of RETTS
Source: PLoS One. 2020 Feb 20;15(2):e0229210. doi: 10.1371/journal.pone.0229210 (PMC7032705; doi:10.1371/journal.pone.0229210)
Supplement: Attachment — Submitted filename: Respons to Reviewers.docx [file pone.0229210.s011.docx]

Lund 29^th^ of January 2020

1

Dear Dr Ehrman,

Thank you for your letter, the comments from you and the reviewers and the opportunity to revise the manuscript *entitled “Scores for sepsis detection and risk stratification – construction of a novel score using a statistical approach and validation of RETTS”*.

The manuscript has now been revised according to the suggestions by you and reviewer 2. Our response to the specific points is given below:

Editors comment:

*While there may not be a strictly "correct" answer, addition of further discussion about why data was assumed to be MAR rather than MNAR and potential limitations and/or ramifications of this decision would strengthen the paper. Can you provide a reference for the sentence in bold below? If so, this would be very nice addition to the paper.*

***“The MAR assumption is not testable, but becomes more reasonable with imputation models that include a wide range of characteristics, including predictors, the outcome and auxiliary variables like in our model”.***

*This additional text could be included in discussion of the limitations.*

Authors reply:

Discussion about limitations of multiple imputation has been added to the discussion, including the text and a reference for the statement that the MAR assumption is not testable, but becomes more reasonable within a model like ours that include several characteristics, including predictors and outcome, p 21, line 428-436 [1].

It is also elaborated in the reply to reviewer 2 below.

Reviewer 2

Reviewers comment 1*: I would like to see a little more addressing the imputation, specifically the R code and more detailed demographic and outcomes data for those patients with missing data, preferably stratified by the type of data element missing. Also, a "worst-case" sensitivity analysis specifically involving lactate (since this was missing so frequently, and directly relates to a major aim), may be worth performing.

I am concerned that little in the edits seem to have substantively addressed the comments regarding imputation. The change added a sentence saying that it was assumed that data was missing at random. I found this unsatisfactory, since a large portion of reviewer and editor feedback raised was very specifically directed at needing to account for the possibility that data was missing not at random.*

Authors reply:

We agree and have made an effort to address the comments on multiple imputation.

First, we have tried to clarify that even though the aims of this study were to develop a sepsis risk stratification tool based on the most predictive, minimal set of vital signs, lactate and HBP plasma levels, this was performed in cohort A, where few values for lactate were missing.

In cohort B the new scores and RETTS were validated and lactate is not included in these scores, supporting information table IV.

To calculate worst case scenario for missing data is an approach for missing data which is a strong assumption that can give the sensitivity analyses a wide range, even with moderate number of missing outcomes.

As mentioned previously, we assumed data to be missing at random, which is not testable, but becomes more reasonable within a model like ours that include several characteristics, including predictors and the outcome.

The cause for that a vital sign or a laboratory value is missing is often that they were considered unnecessary according to the attending physician. The decision was then evidently made based on other available vital parameters and laboratory values and hence the probability of missing depends on other parameters present in the data set; i.e missing at random (Pr (R=0|Yobs, Ymis, ϕ) = Pr(R=0|Yobs,ϕ) [2].

Multiple imputation is therefore commonly used when evaluating clinical risk scores [3-5].

We have elaborated the discussion about limitations of multiple imputation, 21, line 428-436 and added a table on demographics and outcome of patients with missing data to the supporting information (supporting information table V), as well as attached below.

The multiple imputation was performed in SPSS with the code below.

*set the random seed

SET MTINDEX=2000000.

*Analyze Patterns of Missing Values.

MULTIPLE IMPUTATION HR SBP DBP mental_status temperature SaO2

RR oxygen_treatment hbp lactate age

/IMPUTE METHOD=NONE

/MISSINGSUMMARIES OVERALL VARIABLES (MAXVARS=25 MINPCTMISSING=0) PATTERNS.

*Impute Missing Data Values.

DATASET DECLARE newimputeddata.

DATASET DECLARE iterationhistory.

MULTIPLE IMPUTATION HR SBP DBP oxygen_treatment RR

temperature mental_status SaO2 outcome hbp lactate

age sex comorbidities_1 comorbidities_2

comorbidities_3 comorbidities_4 comorbidities_5 comorbidities_6

comorbidities_7

/IMPUTE METHOD=FCS MAXITER= 100 NIMPUTATIONS=20 SCALEMODEL=PMM INTERACTIONS=NONE SINGULAR=1E-012

MAXPCTMISSING=NONE

/CONSTRAINTS age( ROLE=IND)

/CONSTRAINTS hbp ( ROLE=IND)

/CONSTRAINTS sex ( ROLE=IND)

/CONSTRAINTS outcome ( ROLE=IND)

/CONSTRAINTS comorbidities_1( ROLE=IND)

/CONSTRAINTS comorbidities _2( ROLE=IND)

/CONSTRAINTS comorbidities _3( ROLE=IND)

/CONSTRAINTS comorbidities _4( ROLE=IND)

/CONSTRAINTS comorbidities _5( ROLE=IND)

/CONSTRAINTS comorbidities _6( ROLE=IND)

/CONSTRAINTS comorbidities _7( ROLE=IND)

/MISSINGSUMMARIES NONE

/IMPUTATIONSUMMARIES MODELS

/OUTFILE IMPUTATIONS=newimputeddata FCSITERATIONS=iterationhistory.

Supp Table V. Demographics and outcome of patients with missing data.

|  | Cohort A | | | Cohort B | | |  |
| --- | --- | --- | --- | --- | --- | --- | --- |
|  | Without sepsis  n = 28 | | With sepsis  n = 21 | Without sepsis  n=39 | With sepsis  n= 15 | |  |
| Age, median | 57 | 70 | | 70 | | 73 |  |
| Female, n (%) | 12 (43) | 11 (52) | | 23 (59) | | 6 (40) |  |
|  |  |  | |  | |  |  |
| Comorbidities n (%) |  |  | |  | |  |  |
| Diabetes | 1 (4) | 4 (19) | | 8 (21) | | 5 (33) |  |
| Cardiovascular disease | 6 (21) | 7 (33) | | 17 (44) | | 7 (47) |  |
| Renal Disease | 1 (4) | 2 (10) | | 6 (15) | | 4 (27) |  |
| Liver Disease | 1 (4) | 0 (0) | | 0 (0) | | 1 (7) |  |
| Malignancy | 2 (7) | 4 (19) | | 4 (10) | | 2 (13) |  |
| Immunodeficiency | 2 (7) | 1 (5) | | 0 (0) | | 0 (0) |  |
| Respiratory Disease | 2 (7) | 2 (9) | | 6 (15) | | 2 (13) |  |
| No comorbidities | 18 (64) | 8 (38) | | 16 (41) | | 4 (27) |  |
| Organ dysfunction, n (%) |  | 20 (95) | | 22 (56) | | 15 (100) |  |
| Neurologic |  | 2 (9) | | 20 (51) | | 6 (40) |  |
| Cardiovascular |  | 15 (71) | | 14 (36) | | 14 (93) |  |
| Respiratory |  | 3 (14) | | 15 (39) | | 7 (47) |  |
| Renal |  | 5 (24) | | 2 (5) | | 5 (33) |  |
| Hematological |  | 4 (19) | | 2 (5) | | 1 (7) |  |
| Hepatic |  | 3 (14) | | 0 (0) | | 0 (0) |  |
|  |  |  | |  | |  |  |
| Intensive Care n (%) |  | 2 (9) | | 10 (26) | | 3 (20) |  |
| 3-days mortality n (%) |  | 1 (5) | | 3 (8) | | 0 (0) |  |

We feel that the addition of discussion on multiple imputation assumption has improved the manuscript compared to the previous version and hope that you will find it suitable for publication.

Again, thank you for your interest and engagement in our work.

Hoping for a positive response,

Sincerely,

Lisa Mellhammar

References

1. Sterne JA, White IR, Carlin JB, et al. Multiple imputation for missing data in epidemiological and clinical research: potential and pitfalls. BMJ (Clinical research ed.) 2009;**338**:b2393 doi: 10.1136/bmj.b2393[published Online First: Epub Date]|.

2. Buren SV. Flexible Imputation of Missing Data. CRC Press 2018

3. Seymour CW, Kahn JM, Cooke CR, Watkins TR, Heckbert SR, Rea TD. Prediction of critical illness during out-of-hospital emergency care. Jama 2010;**304**(7):747-54 doi: 10.1001/jama.2010.1140[published Online First: Epub Date]|.

4. Seymour CW, Liu VX, Iwashyna TJ, et al. Assessment of Clinical Criteria for Sepsis: For the Third International Consensus Definitions for Sepsis and Septic Shock (Sepsis-3). Jama 2016;**315**(8):762-74 doi: 10.1001/jama.2016.0288[published Online First: Epub Date]|.

5. Sunden-Cullberg J, Rylance R, Svefors J, Norrby-Teglund A, Bjork J, Inghammar M. Fever in the Emergency Department Predicts Survival of Patients With Severe Sepsis and Septic Shock Admitted to the ICU. Crit Care Med 2017;**45**(4):591-99 doi: 10.1097/ccm.0000000000002249[published Online First: Epub Date]|.
